# The social drivers of health for Native Hawaiian and Pacific Islander youth in the United States

**DOI:** 10.1016/j.pmedr.2024.102658

**Published:** 2024-02-17

**Authors:** Reya H. Mokiao, Kristen Carlin, Michael S. Spencer, Bessie A. Young, Amanda M. Fretts

**Affiliations:** a University of Washington School of Medicine, 1959 NE Pacific St, Seattle, WA 98195, USA; b Seattle Children’s Research Institute, 1920 Terry Ave, Seattle, WA 98101, USA; c University of Washington School of Social Work, 4101 15^th^ Ave NE, Seattle, WA 98105, USA; d University of Washington School of Public Health, 3980 15^th^ Ave NE, Seattle, WA 98195, USA

**Keywords:** Indigenous, Native Hawaiian and Pacific Islander, Social determinants of health, Food insecurity, Overweight, Obesity, Children, Adolescents, Youth

## Abstract

**Objectives:**

To describe the social drivers of health and health status of Native Hawaiian and Pacific Islander (NHPI) youth in the US.

**Methods:**

This is a cross-sectional analysis of the 2014 NHPI National Health Interview Survey (NHIS) which surveyed about 3,000 NHPI households, including 1,428 NHPI youth (884 0–12 yo, 421 13–17 yo, and 123 18–21 yo). We described domains of social drivers of health (SDoH), health conditions, and associations of income and food insecurity with body mass index (BMI) for NHPI youth.

**Results:**

NHPI youth come from households with a wide range in income. Approximately 20% of the cohort were food insecure. Among 18–21 yo, 10% report chronic medical conditions (5% with prediabetes). 33% of 13–17 yo and 52% of 18–21 yo were overweight/ obese. For 13–17 yo, lower income was associated with higher BMI. There was no association between food insecurity and BMI for any age group.

**Conclusions:**

Overweight/ obesity are highly prevalent among NHPI youth which is concerning for development of diabetes, hypertension, cardiovascular and kidney disease. Health efforts should focus on SDoH, obesity prevention and management for NHPI youth.

## 1 Introduction

Native Hawaiians and Pacific Islanders (NHPI) have been marginalized and aggregated with other races (Asian) which has made it impossible to understand their health status. However, in 2014, the National Center for Health Statistics conducted the Native Hawaiian and Pacific Islander National Health Interview Survey (NHPI NHIS)—the first federal survey to measure the health and social drivers of NHPI in the US, which has not been repeated. The purpose of this study was to describe the social drivers of health (SDoH) and health measures of NHPI youth (0–21 year old (yo)) based on the 2014 NHPI NHIS.

## 2 Methods

The NHIS is a cross-sectional survey to monitor the nation’s health by collecting information related to health status, conditions, access to health services, and risk factors. The NHIS queries several domains of the SDoH, including education, access and use of healthcare, and economic stability. In 2014, NHIS surveyed about 3,000 NHPI households, including 1,428 NHPI youth (884 0–12 yo, 421 13–17 yo and 123 18–21 yo), which was the last year of intentional NHPI participant inclusion. We included 18–21 yo to highlight social drivers and health status of this age group to increase awareness for pediatric providers, who often see patients until 21 years old. Food security status was assessed via the USDA 10 question family food security supplement. Details on NHPI NHIS are published elsewhere (National Center for Health Statistics, 2017). NHPI NHIS is publicly available anonymized data and thus exempt from ethical compliance. To obtain population-based estimates, weighted frequencies and percentages were calculated for categorical variables and weighted means were calculated for continuous variables. Analyses were completed separately for 0–12, 13–17 and 18–21 yo. Logistic and linear regression models to explore associations of food insecurity and income with body mass index (BMI) were performed for 13–17 yo and 18–21 yo, adjusted for demographics. We excluded participants with missing values and complete case analysis was performed. Spearman correlation coefficients over 0.5 and variance inflation factor (VIF) values over 5 were used to identify issues with multicollinearity (Vatcheva et al., 2016). A p-value of 0.05 was considered statistically significant. SAS 9.4 (Cary, NC) survey procedures (PROC SURVEYMEANS, PROC SURVEYFREQ, PROC SURVEYLOGISTIC, and PROC SURVEYREG) were used for all analyses to account for the complex survey design.

## 3 Results

The education, economic stability, and healthcare domains of the SDoH are shown in Table 1. Approximately 30 % of NHPI youth came from households with a bachelor’s degree or higher. Many NHPI children received care from a clinic and had seen a doctor in the past year, but only half of 18-21yo were seen by a doctor in the past year. There was a wide range in household income, but it’s notable that approximately 29–44 % of NHPI youth came from homes with low incomes of less than $50,000/yr. About 20 % of NHPI youth were food insecure (low and very low food security). Among 18–21 yo, 12 (10 %) had self-reported chronic medical conditions: 5 % with prediabetes and 5 % with hypertension, kidney disease, high cholesterol, coronary heart disease, and/or diabetes (See Table 1). In total, 33 % of 13–17 yo and 52 % of 18–21 yo were overweight or obese. Associations of income and food insecurity with BMI are shown in Table 2 (See supplemental tables for estimates and OR of model covariates). Compared to 13–17 yo with annual household income ≥ $100,000, 13–17 yo with annual household income $50,000–99,999 had 0.97 kg/m^2^ higher BMI, and those with annual household income < $50,000 had 1.30 kg/m^2^ higher BMI. 18–21 yo had a similar pattern with decreasing income and increasing BMI; this relationship was not statistically significant perhaps due to small sample size. There was no association with income and categorical BMI overweight/obesity for any age group. Food insecurity was not associated with BMI for any age group, consistent with findings from a recent analysis of NHPI NHIS by Long et al. (2023).

**Table 1:** The social drivers of health and health measures for NHPI youth from the 2014 NHPI NHIS stratified by age group.

**Characteristic**	**0**–**12 yo**	**13**–**17 yo**	**18**–**21 yo**
**Participants, n**	884	421	123
**Age, mean (SE), y**	6 (0.2)	15 (0.1)	19 (0.2)
**Sex, n (%)**			
Male	455 (51)	203 (51)	66 (50)
Female	429 (49)	218 (49)	57 (50)
**Education^a^, n (%)**			
12th grade or less	33 (4)	7 (3)	2 (1)
High school/ GED	200 (27)	91 (25)	28 (26)
Some college, no degree	198 (23)	100 (25)	31 (27)
Associate degree	169 (16)	69 (16)	25 (20)
Bachelor's degree	177 (18)	99 (19)	28 (17)
Master's, Professional and/or Doctoral degree	107 (12)	55 (12)	9 (9)
**Economic Stability**			
Income ^b^, n (%)			
≥ $100,000	236 (24)	120 (25)	32 (26)
$50,000–99,999	300 (37)	153 (46)	34 (30)
$0–49,999	287 (39)	112 (29)	51 (44)
SNAP/ EBT^c^, n (%)	243 (33)	78 (23)	26 (27)
WIC^c^, n (%)	171 (24)	15 (9)	8 (7)
Food Security Status, n (%)			
High food security	602 (67)	313 (71)	87 (71)
Marginal food security	115 (13)	43 (12)	10 (7)
Low food security	108 (14)	39 (8)	14 (11)
Very low food security	59 (6)	26 (9)	12 (11)
Problems paying medical bills, n (%)	167 (22)	85 (21)	22 (17)
**Healthcare**			
Health insurance^d^, n (%)	868 (98)	415 (98)	117 (94)
Care from clinic/ doctor's office, n (%)	842 (96)	392 (96)	–
WCC/ general doctor in past yr, n (%)	757 (85)	326 (77)	70 (55)
**BMI category^e^, n (%)**			
Underweight	–	7 (2)	5 (6)
Normal weight	–	236 (65)	55 (42)
Overweight	–	63 (19)	27 (22)
Obese	–	44 (14)	36 (30)
**Health Conditions, n (%)**			12 (10)
Hypertension only, n (%)	–	–	2 (2)
Hypertension & diabetes, n (%)	–	–	1 (1)
Hypertension & high cholesterol, n (%)	–	–	1 (1)
Weak/ failing kidneys, n (%)	–	–	1 (<1)
Coronary heart disease, n (%)	–	–	1 (<1)
Prediabetes, n (%)	–	–	6 (5)
**Smoking Status, n (%)**			
Current smoker	–	–	15 (11)
Never smoker	–	–	106 (88)
Former smoker			2 (1)
Ever used e-cig, n (%)	–	–	37 (25)

n is unweighted, proportions are weighted, SDoH = social drivers of health, SE = standard error, a. Highest level of education attained by adult family member, b. Total annual combined family income, SNAP = supplemental nutrition assistance program, EBT = electronic benefit transfer, WIC = Special Supplemental Nutrition Program for Women, Infants, and Children, c. Use by a family member, WCC = well child check, d. Family member with health insurance, e. Based on standard categories for body mass index (BMI), - significant missing or information not obtained. Note that the sample size for each variable estimate may vary depending on missingness.

Data Source: NCHS, Native Hawaiian and Pacific Islander National Health Interview Survey, 2014. (National Center for Health Statistics, 2014)

**Table 2:** Associations of income and food insecurity with BMI (continuous and categorical) among NHPI youth from the 2014 NHPI NHIS.

		BMI (continuous)				BMI (categorical overweight/obese)			
		13–17 yo		18–21 yo		13–17 yo		18–21 yo	
		β (95% CI)	p	β (95% CI)	p	OR (95% CI)	p	OR (95% CI)	p
Model 1	Income 0 -$49,999	0.96 (0.01, 1.90)	0.05	1.82 (-3.42, 7.06)	0.48	0.82 (0.34, 1.85)	0.62	2.18 (0.60, 7.86)	0.22
	Income $50,000–99,999	0.87 (-0.01, 1.75)	0.05	1.22 (-3.83, 6.28)	0.62	1.83 (0.86, 3.89)	0.11	1.56 (0.44, 5.45)	0.48
	Food Insecure	−0.74 (-1.97, 0.49)	0.23	0.48 (-4.06, 5.02)	0.83	0.88 (0.36, 2.15)	0.77	1.17 (0.23, 5.92)	0.84
Model 2	Income 0 -$49,999	1.30 (0.27, 2.33)	0.02*	2.55 (-1.63, 6.73)	0.22	1.24 (0.49, 3.14)	0.64	2.94 (0.91, 9.57)	0.07
	Income $50,000–99,999	0.97 (0.08, 1.85)	0.03*	0.91 (-4.50, 6.35)	0.73	2.09 (0.99, 4.41)	0.05	1.45 (0.40, 5.31)	0.56
	Food Insecure	−0.54 (-1.75, 0.67)	0.37	0.92 (-3.44, 5.28)	0.67	1.11 (0.38, 3.23)	0.84	1.35 (0.31, 5.88)	0.68

Model 1: adjusted for age, sex, education level

Model 2: Model 1 + supplemental food assistance (SNAP or WIC use)

For all models, reference for income is ≥ $100,000/yr or greater and reference for food insecure is food secure

Education variable is 3 levels 1) high school graduate/ GED recipient or less, 2) some college, no degree/ Associate’s degree, 3) Bachelor’s degree or higher (reference)

BMI categorical reference group is non-overweight/obese (normal or underweight)

*Statistically significant p < 0.05

Data Source: NCHS, Native Hawaiian and Pacific Islander National Health Interview Survey, 2014. (National Center for Health Statistics, 2014)

## 4 Discussion

This is the first description of the SDoH of NHPI youth in the US. Food insecurity among NHPI youth was higher than all children in the US (12.5 %), but comparable to Black (22 %) and Latino children (18.5 %) (Coleman-Jensen et al., 2022). About 33 % of 13–17 yo and 52 % of 18–21 yo were overweight or obese which suggests that childhood may be a critical time for obesity prevention. The high prevalence of overweight and obesity is concerning for development of chronic diseases such as diabetes, hypertension, cardiovascular disease and kidney disease. Approximately 10 % of 18–21 yo report a chronic medical condition with 5 % with prediabetes which may be underestimated given only half had seen a doctor in the past year. We found lower income was associated with higher BMI; this has been reported previously in the literature (Kim and von dem Knesebeck, 2018), but needs further exploration including the role of diet, social drivers such as neighborhood and family structure and environmental factors. Limitations of this study include inability to determine causality as this is a cross-sectional survey and bias in self-report of SDoH and medical conditions. Participants 18–21 yo were included to increase awareness for pediatric providers. However, information gathered for 18–21 yo differed from those younger than 18 yo which introduces data quality discrepancies; to mitigate, data was stratified by age group and analyzed separately. The NHPI NHIS was issued in English which limits participation of non-English speakers, but outreach materials had expressions of greetings and thanks in over 20 NHPI languages. The NHPI NHIS public use data file does not include access to state or geographic identifiers or Pacific Island of origin due to risk of inadvertent disclosure of confidential information. Despite these limitations, this national survey provides the best snapshot of NHPI youth in the US and illustrates that more work and evaluation needs to be done for this high risk population. Other domains and measures of the SDoH and their role in BMI and chronic disease should also be explored to inform future preventative efforts for NHPI youth especially given the high prevalence of obesity, diabetes, hypertension and kidney disease among NHPI adults (Galinsky et al., 2014).

## 5 Conclusion

The high prevalence of overweight/ obesity among NHPI youth is alarming especially since overweight/ obesity increases the risk for many chronic medical conditions such as diabetes, hypertension, cardiovascular disease, kidney disease and cancer to name a few. The future of health and health equity for NHPI people must focus on health of the youth. Interventions and resources for overweight/ obesity prevention and management should be provided for NHPI youth and families especially for those from low-income households.

This manuscript was presented as a poster at the National Institute of Diabetes and Digestive and Kidney Diseases (NIDDK) Network of Minority Health Research Investigators (NMRI) Workshop in Bethesda, MD on April 20, 2023.

## Funding

This research did not receive any specific grant from funding agencies in the public, commercial, or not-for-profit sectors.

## CRediT authorship contribution statement

**Reya H. Mokiao:** Writing – review & editing, Writing – original draft, Supervision, Methodology, Investigation, Conceptualization. **Kristen Carlin:** Writing – review & editing, Methodology, Formal analysis. **Michael S. Spencer:** Writing – review & editing, Supervision, Investigation, Conceptualization. **Bessie A. Young:** Writing – review & editing, Supervision, Methodology, Investigation, Conceptualization. **Amanda M. Fretts:** Writing – review & editing, Supervision, Methodology, Investigation, Conceptualization.

## Declaration of competing interest

The authors declare that they have no known competing financial interests or personal relationships that could have appeared to influence the work reported in this paper.

## Data Availability

Data will be made available on request.
